# High sensitivity and negative predictive value of the DETECT algorithm for an early diagnosis of pulmonary arterial hypertension in systemic sclerosis: application in a single center

**DOI:** 10.1186/s13075-017-1327-8

**Published:** 2017-06-14

**Authors:** Alfredo Guillén-Del Castillo, Eduardo L. Callejas-Moraga, Gabriela García, José F. Rodríguez-Palomares, Antonio Román, Cristina Berastegui, Manuel López-Meseguer, Enric Domingo, Vicente Fonollosa-Plá, Carmen Pilar Simeón-Aznar

**Affiliations:** 1 Department of Systemic Autoimmune Diseases, Hospital Universitari Vall d’Hebron, Universitat Autònoma de Barcelona, Passeig Vall d’Hebron 119-129, PC: 08035 Barcelona, Spain; 2 Department of Cardiology, Hospital Universitari Vall d’Hebron, Universitat Autònoma de Barcelona, Barcelona, Spain; 3 Department of Pneumology, Hospital Universitari Vall d’Hebron, Universitat Autònoma de Barcelona, Barcelona, Spain

**Keywords:** Systemic sclerosis, Pulmonary arterial hypertension, Echocardiography, Screening tools

## Abstract

**Background:**

Pulmonary arterial hypertension (PAH) is one of the most relevant causes of death in systemic sclerosis. The aims of this study were to analyse the recently published DETECT algorithm comparing it with European Society of Cardiology/European Respiratory Society (ESC/ERS) 2009 guidelines: as screening of PAH; (2) identifying median pulmonary arterial pressure (mPAP) ≥21 mmHg; and (3) determining any group of pulmonary hypertension (PH).

**Methods:**

Eighty-three patients fulfilling LeRoy’s systemic sclerosis diagnostic criteria with at least right heart catheterization were studied retrospectively. Clinical data, serological biomarkers, echocardiographic and hemodynamic features were collected. SPSS 20.0 was used for statistical analysis.

**Results:**

According to right heart catheterization findings, 35 patients with PAH and 28 with no PH met the standards for DETECT algorithm analysis: 27.0% of patients presented with functional class III/IV. Applying DETECT, the sensitivity was 100%, specificity 42.9%, the positive predictive value 68.6% and the negative predictive value 100%, whereas employing the ESC/ERS guidelines these were 91.4%, 85.7%, 88.9% and 89.3%, respectively. There were no missed diagnoses of PAH using DETECT compared with three patients missed (8.5%) using ESC/ERS guidelines. The DETECT algorithm also showed greater sensitivity and negative predictive value to identify patients with mPAP ≥21 mmHg or with any type of PH.

**Conclusions:**

The DETECT algorithm is confirmed as an excellent screening method due to its high sensitivity and negative predictive value, minimizing missed diagnosis of PAH. DETECT would be accurate either for early diagnosis of borderline mPAP or any group of PH.

## Background

Systemic sclerosis (SSc) is an autoimmune disease characterized by inflammation and autoimmunity, aberrant tissue reparation with excessive extracellular matrix deposition and altered vascular regeneration and endothelial injury [1, 2]. Pulmonary arterial hypertension (PAH) is one of the most severe complications in patients with SSc, in whom the prevalence is 10% as diagnosed by right heart catheterization (RHC) [3, 4]. PAH has a major negative impact on survival and has become one of the leading causes of SSc-related deaths [5–7]. The outcome of this disease unfortunately is poor, with a 51% survival rate at 5 years of diagnosis even after the introduction of potent vasodilatator agents [8]. Despite SSc being a well-known risk factor for developing PAH, there are still delays in diagnosing PAH and consequently more than 80% of patients present with World Health Organization (WHO) functional class III or IV at this point [8].

A French nationwide prospective multicentre study aimed to compare early PAH diagnosis using Doppler echocardiography prior to referral for RHC compared with routine clinical practice [9]. Later, the authors demonstrated prompt PAH identification in patients with lower functional classes of disease and subsequently a fourfold superior survival ratio [10]. The European Society of Cardiology and the European Respiratory Society (ESC/ERS) guidelines published in 2009 [11] recommended echocardiography in symptomatic patients. RHC was carried out if the tricuspid regurgitant velocity (TRV) was ≥3.4 m/s, TRV between 2.8-3.4 m/s with symptoms or TRV ≤2.8 m/s with symptoms plus additional echocardiographic variables suggestive of PH.

Recently, Coghlan et al. reported the first evidence-based algorithm for the screening of PAH in SSc (DETECT) [12]. The DETECT algorithm is a tool to identify patients with PAH in the asymptomatic stages, through the study of clinical variables, pulmonary function tests, immunological, biological, electrocardiographic and finally echocardiographic parameters. The authors demonstrated higher sensitivity of PAH detection than that achieved using the ESC/ERS 2009 guidelines.

The main aim of this study was to determine the value of the DETECT algorithm in a different population of patients with SSc from that studied in the original work and to compare it with the ESC/ERS 2009 guidelines. Other objectives were to ascertain the ability of DETECT to predict median pulmonary arterial pressure (mPAP) ≥21 mmHg or to predict patients with pulmonary hypertension (PH) versus patients with no PH.

## Methods

### Patients

Eighty-three patients with SSc who had undergone at least one RHC were studied retrospectively. The RHC was indicated due to suspicion of PAH after undergoing an annual complete pulmonary function test and annual echocardiography as routine monitoring of SSc. The reasons for carrying out the RHC in our population were the presence of right ventricular systolic pressure (RVSP) >36 mmHg plus a forced vital capacity/diffusing capacity for carbon monoxide (FVC/DLCO) ratio >1.6 in 35 patients, 34 patients had RVSP >36 mmHg, 8 patients had a FVC/DLCO ratio >1.6 and 6 patients suffered exclusively from progressive unexplained dyspnoea. All patients fulfilled LeRoy’s SSc diagnostic criteria [13], and 80 patients also met the American College of Rheumatology/European League Against Rheumatism (ACR/EULAR) 2013 classification criteria [14]. The duration of disease was more than 3 years from the first non-Raynaud’s phenomenon symptom in all cases. For validation of the DETECT algorithm, four patients were excluded as they presented with a FVC lower than the 40% predicted value, and another patient was excluded who did not meet the inclusion criteria due to DLCO ≥60%.

### Comparisons of the DETECT algorithm and the ESC/ERS 2009 guidelines

**Fig. 1:**
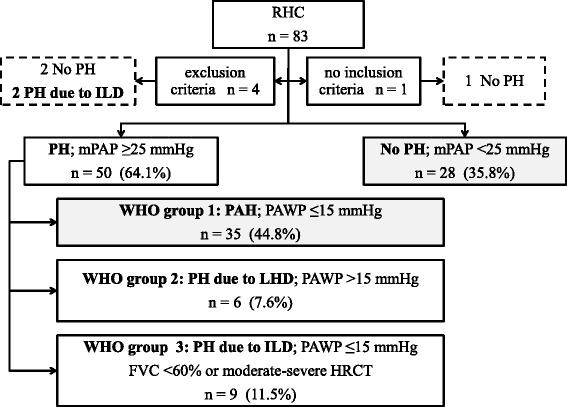
Flow of patients in the study population. Eighty-three patients with systemic sclerosis had at least one right heart catherization (*RHC*), which was revised retrospectively. Four patients were excluded due to the exclusion criteria (all patients had forced vital capacity (*FVC*) <40%), and another patient was excluded due to not meeting the inclusion criteria, who had a diffusing capacity for carbon monoxide (DLCO) ≥60%. Patients with pulmonary arterial hypertension (*PAH*) and patients without pulmonary hypertension (*no PH*) (*n* = 63) were selected for the following studies: (1) validation of the DETECT algorithm for screening of PAH and (2) identification of patients with median pulmonary arterial pressure (mPAP) ≥21 mmHg and (3) determination of any group of PH (all patients (*n* = 83) were included in this study). *WHO* World Health Organization, *PAWP* pulmonary artery wedge pressure, *LHD* left heart disease, *ILD* interstitial lung disease, *HRC*T high-resolution computed tomography

Three different scenarios were conducted for the comparison of the DETECT algorithm with the ESC/ERS 2009 guidelines: First, 35 patients diagnosed with PAH and 28 with no PH after undergoing RHC were selected for the application of the DETECT algorithm (see Fig. 1). Second, these 63 patients were used to explore the capacity of both screening tests to identify mPAP ≥21 mmHg (both borderline mPAP and PAH). Third, with neither inclusion nor exclusion criteria 52 patients diagnosed with PH and 31 with no PH were selected to investigate the detection of any sort of PH.

### Data collection

Data from the first available RHC of each patient were revised. PH was defined as mPAP ≥25 mmHg in RHC, and was classified in the following groups: WHO group 1 or PAH was defined if pulmonary artery wedge pressure (PAWP) was ≤15 mmHg and pulmonary vascular resistance (PVR) was >3 Wood units in RHC [15]; WHO group 2 or PH due to left heart disease, if PAWP was >15 mmHg; WHO group 3 or PH due to interstitial lung disease (ILD) if PAWP was ≤15 mmHg and predicted FVC <60% or if the extent of interstitial disease was identified as moderate-severe on high-resolution computed tomography (HRCT) [12]. Borderline mPAP (BoPAP) was considered if mPAP was between 21 and 24 mmHg, with PAWP ≤15 mmHg and if there was no ILD or the FVC was >60% [16].

Based on the extent of skin involvement, patients were divided into those with diffuse cutaneous SSc (dcSSc) if thickening of the skin was distal and proximal to the elbows or knees, limited cutaneous SSc (lcSSc) if the affected skin did not exceed this limit proximally and sine scleroderma systemic sclerosis (ssSSc) if no skin thickening was present [17]. The disease onset was described as the date of the first symptom attributable to SSc, including Raynaud’s phenomenon (RP), although the time from the first symptom excluding RP also was analysed. ﻿All data were collected at the time of RHC. 

The presence of telangiectasias, past or current digital ulcers (DU) and past history of scleroderma renal crisis (SRC) was recorded. ILD was defined as radiological evidence of interstitial disease on HRCT. Cardiac involvement was defined as current or past history of pericardial effusion, left ventricular ejection fraction (LVEF) <50% and ischaemic heart disease with no cardiovascular risk factor or conduction abnormalities.

Antinuclear antibodies (ANA) were evaluated by indirect immunofluorescence (IIF) assay using HEp-cell line 2. Anticentromere antibodies (ACA) were described by IIF or commercial line blot assay to centromere proteins A and B (EUROLINE Systemic Sclerosis (Nucleoli) Profile (IgG), Euroimmun, Germany). Anti-topoisomerase I antibodies were determined by enzyme-linked immunosorbent assay.

Demographics, cardiovascular risk factors, anthropometrical data, complete pulmonary function test (PFT), 6-minute walking distance (6MWD), WHO-functional class, laboratory data including serum urate, N-terminal pro-brain natriuretic peptide (NT-proBNP) and right axis deviation in electrocardiography (ECG) were registered. Echocardiography included the determination of left ventricular ejection fraction (LVEF), right atrium (RA) area, tricuspid annular plane systolic excursion (TAPSE), tricuspid regurgitation velocity (TRV) and RVSP.

RHC details consisted of mPAP, PAWP, right atrial pressure (RAP), transpulmonary pressure gradient (TPG), cardiac output (CO), pulmonary vascular resistance (PVR), systemic vascular resistance (SVR), mixed venous oxygen saturation (SvO2) measured in pulmonary artery and arterial oxygen saturation (SaO2) measured in arterial blood gas analysis.

The application of the DETECT algorithm was conducted in two steps through the website PAH risk calculator (http://detect-pah.com) [18], the first step for referring the patient for echocardiography and the second for carrying out RHC [12]. The indication of RHC following ESC/ERS 2009 guidelines was considered if: (1) TRV was >3.4 m/s; or (2) TRV was >2.8 to **≤**3.4 m/s, with symptoms (undue dyspnoea or fatigue, chest pain, near syncope or signs of right heart failure); or (3) TRV was **≤**2.8 m/s, with symptoms and additional echocardiographic variables suggestive of PH.

### Statistical analysis

Qualitative data were expressed as the mean and standard deviation (SD) after checking the data using the normal distribution test, and non-normally distributed qualitative variables were described as the median and interquartile range (IQR). To assess whether there were statistically significant differences, Student’s *t* test or the Mann-Whitney *U* test were used according to the result of the normal distribution test. Categorical variables were analysed by the chi-square test and Fisher’s exact test. The 95% confidence intervals (CIs) were calculated for sensitivity, specificity, positive predictive value (PPV) and negative predictive value (NPV). A *p* value <0.05 was considered significant. Statistical analysis was conducted using SPSS 20.0 for Windows (SPSS Inc., Chicago, IL, USA).

## Results

### Baseline characteristics

Comparing the indications for RHC in 83 patients, the proportions of patients diagnosed with PH were 24/35 patients (68.5%) with RVSP >36 mmHg plus FVC/DLCO ratio >1.6, 23/34 patients (67.6%) with RVSP >36 mmHg, 4/8 patients (50.0%) with FVC/DLCO ratio >1.6 and 1/6 patients (16.6%) with progressive unexplained dyspnoea. Two the four patients who were excluded had PH due to ILD. Following the RHC results in the 78 patients who fulfilled the inclusion criteria and did not meet the exclusion criteria, PH was identified in 50 patients (64.1%) and PH was ruled out in the other 28 subjects (35.8%), although 14 of them had BoPAP with mPAP between 21 and 24 mmHg (Fig. 1). According to PH classification, 35 patients (44.8%) were diagnosed with PAH, 6 (7.6%) had PH due to left heart disease and 9 (11.5%) had PH due to ILD. Sixty-three patients with PAH and without PH were selected for DETECT algorithm analysis.

The baseline characteristics of the selected patients with SSc are summarized in Table 1. There were 59 women and 4 men, the mean ± SD age at RHC was 62.4 ± 11.6 years. Based on skin involvement, lcSSc was the most frequent cutaneous subtype in 45 patients (71.4%), 10 patients (15.9%) had dcSSc and 8 patients (12.7%) had ssSSc. The mean time from the first SSc symptom including RP was 18.6 ± 12.3 years, while for the time from first non-RP it was 10.5 ± 8.9 years. Almost the whole study population (*n* = 61 (96.8%)) fulfilled the ACR/EULAR 2013 classification criteria. There were no statistical differences in the presence of telangiectasias, past or current DU, history of SRC, ILD or cardiac involvement, when comparing the two groups. Although there were no statistical differences in positivity to antinuclear antibodies or the observation of a nucleolar pattern in the indirect immunofluorescence, patients with PAH more often had ACA (*n* = 23, 65.7% vs *n* = 11, 39.3%, *p* = 0.03), but lower positivity against anti-topoisomerase I antibodies (*n* = 3, 8.6% vs *n* = 9, 32.1%, *p* = 0.01). Arterial hypertension was the most frequent cardiovascular risk factor in the whole group, being identified in 27 patients (42.9%), followed by dyslipidaemia in 15 patients (23.8%); however, there were no patients with diabetes mellitus. No statistical differences were identified in the anthropometrical variables. The patients with PAH received less immunosuppressant therapy than the control group, and less treatment with calcium channel blockers. However, there were no statistical differences in the use of specific vasodilator therapy, for which the indication was RP in all cases.

**Table 1:** Demographic data and baseline characteristics

	All patients (*n* = 63)	PAH patients (*n* = 35)	No PH patients (*n* = 28)	*P* value
Female^a^	59 (93.7)	34 (97.1)	25 (89.3)	0.31
Age, years^b^	62.4 (±11.6)	64.4 (± 10.8)	59.9 (± 12.4)	0.13
DcSSc^a^	10 (15.9)	4 (11.4)	6 (21.4)	0.31
Time from first symptom, years^b^	18.6 (± 12.3)	19.6 (±11.2)	17.2 (± 13.7)	0.29
Time from first non-RP symptom, years^b^	10.5 (± 8.9)	9.7 (±9.4)	11.5 (± 8.2)	0.21
ACR/EULAR 2013 classification criteria^a^	61 (96.8)	34 (97.1)	27 (96.4)	1.0
Telangiectasias^a^	59 (93.7)	33 (94.3)	26 (92.9)	1.0
Digital ulcers^a^	35 (55.6)	20 (57.1)	15 (53.6)	0.77
Scleroderma renal crisis^a^	2 (3.2)	2 (5.7)	0 (0)	0.49
Interstitial lung disease^a^	32 (50.8)	19 (54.3)	12 (42.9)	0.45
Cardiac involvement^a^	26 (41.3)	17 (48.6)	9 (32.1)	0.18
Antinuclear antibodies^a^	62 (98.4)	34 (97.1)	28 (100)	1.0
ACA^a^	34 (54.0)	23 (65.7)	11 (39.3)	0.03
Anti-Scl-70^a^	12 (19.0)	3 (8.6)	9 (32.1)	0.01
Nucleolar IIF pattern^a^	4 (6.3)	3 (8.6)	1 (3.6)	0.62
Arterial hypertension^a^	27 (42.9)	14 (40)	13 (43.4)	0.60
Dyslipidaemia^a^	15 (23.8)	6 (17.1)	9 (32.1)	0.16
Diabetes mellitus^a^	0 (0)	0 (0)	0 (0)	NA
Immunosuppressant therapy^a^	25 (39.7)	8 (22.9)	17 (60.7)	<0.01
Prednisone^a^	19 (30.2)	7 (20.0)	12 (40.9)	0.04
Sodium mycophenolate^a^	7 (11.1)	1 (2.9)	6 (21.4)	0.03
Azathioprine	3 (4.8)	0 (0)	3 (10.7)	0.08
IV cyclophosphamide^a^	12 (19.0)	3 (8.6)	9 (32.1)	0.01
Calcium channel blocker^a^	33 (52.4)	14 (40.0)	19 (67.9)	0.02
Specific vasodilator therapy^a^	13 (20.6)	7 (20.0)	6 (21.4)	0.88
IV prostanoids^a^	1 (1.6)	1 (2.9)	0 (0)	1.0
ERA^a^	10 (15.9)	5 (14.3)	5 (17.9)	0.74
PDE5 I^a^	5 (7.9)	2 (5.7)	3 (10.7)	0.64

*PAH* pulmonary arterial hypertension, *PH* pulmonary hypertension, *dcSSc* diffuse cutaneous systemic sclerosis, *ACR/EULAR* American College of Rheumatology/European League Against Rheumatism, *RP* Raynaud’s phenomenon, *ACA* anticentromere antibodies, *IIF* indirect immunofluorescence, *NA* not applicable, *IV* intravenous, *ERA* endothelin receptor antagonist, *PDE5 I* phosphodiesterase type-5 inhibitor. ^a^Data are shown as number (%) for categorical variables. ^b^Data are shown as mean (± SD)

Regarding respiratory parameters, the PAH group had higher FVC percent predicted/DLCO percent predicted than controls (2.0 ± 0.7% vs 1.5 ± 0.4%, *p* < 0.01) and shorter 6MWD (232.8 ± 76.7 m vs 322.4 ± 105.0 m, *p* = 0.01) (Table 2). Advanced WHO functional classes III/IV were more common in patients with PAH (*n* = 15 (42.9%)), than in the group with no PH (*n* = 2 (7.1%)) (*p* < 0.01). There were higher NT-proBNP and serum urate levels and higher prevalence of right axis deviation on ECG in the PAH group. In relation to echocardiographic data, patients with PAH had a greater RA area; moreover, they presented with higher TRV and RVSP than patients with no PH. Therefore, there were statistically significant differences between PAH and without PH patients in seven out of eight variables considered in DETECT algorithm.

**Table 2:** Respiratory, laboratory and echocardiography data

	All patients (*n* = 63)	PAH patients (*n* = 35)	No PH patients (*n* = 28)	*P* value
Pulmonary function tests				
FVC, % predicted^b^	75.2 (± 16.4)	75.6 (± 15.5)	74.7 (± 17.8)	0.86
DLCO, % predicted^b^	46.3 (± 14.2)	41.5 (± 13.9)	51.0 (± 13.1)	0.01
FVC%/DLCO%^b^	1.8 (± 0.6)	2.0 (± 0.7)	1.5 (± 0.4)	<0.01
6MWD, m^b^	256.5 (± 92.5)	232.8 (± 76.7)	322.4 (± 105.0)	0.01
WHO functional class III/IV^a^	17 (27.0)	15 (42.9)	2 (7.1)	<0.01
Laboratory				
NT-proBNP, pg/mL^c^	198 (41 to 647)	1271 (580 to 3154)	87 (32 to 202)	<0.001
Serum urate, mg/dL^b^	5.6 (± 1.9)	6.8 (± 1.9)	4.6 (± 1.0)	<0.001
Right axis in ECG^a^	18 (28.6)	10 (28.6)	1 (3.6)	0.01
Echocardiography				
LVEF, %^c^	60.0 (59.0 to 65.0)	60.0 (58.5 to 65.0)	62.0 (59.7 to 65.0)	0.43
TAPSE, mm^b^	19.4 (± 3.9)	19.1 (± 4.6)	19.8 (± 3.0)	0.55
RA area, cm2^c^	16.0 (13.0 to 21.0)	17.0 (15.0 to 22.7)	14.0 (12.0 to 16.0)	<0.01
TRV, m/s^b^	3.5 (± 0.7)	4.0 (± 0.6)	2.8 (± 0.3)	<0.001
RVSP, mmHg^c^	59.5 (44.0 to 76.2)	73.0 (61.0 to 85.0)	44.0 (37.0 to 51.0)	<0.001

*PAH* pulmonary arterial hypertension, *PH* pulmonary hypertension, *FVC* forced vital capacity, *DLCO* diffusing capacity for carbon monoxide, *6MWD* 6-minute walking distance, *WHO* World Health Organization, *NT-proBNP* N-terminal pro-brain natriuretic peptide, *ECG* electrocardiography, *LVEF* left ventricular ejection fraction, *TAPSE* tricuspid annular plane systolic excursion, *RA* right atrium, *TRV* tricuspid regurgitant velocity, *RVSP* right ventricular systolic pressure. ^a^Data are shown as number (%) for categorical variables. ^b^Data are shown as mean (± SD). ^c^Data are shown as median (IQR)

RHC in the PAH group was characterized by superior mean PAP (Table 3). Specifically, the median (IQR) of mPAP was 42.0 (33.0 − 50.0) mmHg in patients with PAH, while it was 20.5 (17.0 to 23.0) mmHg in the group with no PH (*p* < 0.001). Furthermore, TPG was increased in patients with PAH, but CO was lower in those patients. The PAH group had higher pulmonary vascular resistance, with lower SvO2 and SaO2 during the RHC, compared with the group with no PH.

**Table 3:** Haemodynamic characteristics on right heart catheterization

	All patients (*n* = 63)	PAH patients (*n* = 35)	No PH patients (*n* = 28)	*P* value
Mean PAP, mmHg^b^	31.0 (21.0 to 44.0)	42.0 (33.0 to 50.0)	20.5 (17.0 to 23.0)	<0.001
PAWP, mmHg^a^	9.6 (± 4.0)	9.7 (± 4.8)	9.5 (± 3.4)	0.90
RAP, mmHg^b^	5.0 (2.5 to 8.5)	5.0 (3.0 to 10.0)	4.5 (2.0 to 7.0)	0.14
TPG, mmHg^a^	20.0 (± 13.4)	31.4 (± 11.8)	10.6 (± 3.9)	<0.001
Cardiac output, L/min^b^	3.5 (2.9 to 4.5)	3.4 (2.8 to 4.0)	3.9 (2.9 to 5.1)	0.05
PVR, WU^a^	6.6 (± 4.8)	9.9 (± 4.6)	3.2 (± 1.6)	<0.001
SVR, WU^a^	26.9 (± 10.3)	28.6 (± 9.7)	25.2 (± 11.0)	0.42
SvO2, %^a^	66.3 (± 8.1)	61.8 (± 8.3)	71.0 (± 4,7)	<0.001
SaO2, %^a^	93.5 (± 4.0)	91.2 (± 3.7)	96.1 (± 2.5)	<0.001

*PAH* pulmonary arterial hypertension, *PH* pulmonary hypertension, *PAP* pulmonary artery pressure, *PAWP* pulmonary artery wedge pressure, *RAP* right atrial pressure, *TPG* transpulmonary pressure gradient, *PVR* pulmonary vascular resistance, *WU* Wood units, *SVR* systemic vascular resistance, *SvO2* mixed venous oxygen saturation, *SaO2* arterial oxygen saturation. ^a^Data are shown as mean (± SD). ^b^Data are shown as median (IQR)

### Comparisons of the DETECT algorithm and the ESC/ERS 2009 guidelines

The DETECT algorithm steps are represented in Table 4. After step 1 of the algorithm 35 patients in the PAH group (100%) would have been referred to echocardiography compared to 24 in the group without PH (85.7%) (*p* = 0.03). Following the echocardiography results, RHC would have been recommended in all 35 patients in the PAH group (100%), while this was only the case in 16 subjects (57.1%) in patients with no PH (*p* < 0.001). However, based on the ESC/ERS 2009 guidelines, 32 patients with PAH (91.4%) and 4 patients in the group without PH (14.3%) would have been referred for RHC. The RHC referral ratio was higher using DETECT than it was using the ESC/ERS guidelines. The main reasons for not referring these patients for RHC using the ESC/ERS 2009 guidelines would have been that 12 patients had TRV >2.8 to **≤**3.4 m/s but with no symptoms, 5 patients presented with TRV **≤**2.8 m/s with symptoms but with no additional echocardiographic signs of PH, and another 10 patients had TRV **≤**2.8 m/s with no symptoms and no additional echocardiographic signs of PH. There would have been no missed diagnosis of PAH with DETECT, but using the ESC/ERS guidelines it would have reached 8.5% (Table 5). The sensitivity (95% CI) of the DETECT algorithm was 100% (90.1–100) compared to sensitivity of 91.4% (77.6–97.0) using the ESC/ERS 2009 guidelines. The specificity was lower with DETECT at 42.9% (26.5–60.9) whereas it was 85.7% (68.2–94.3) using the ESC/ERS 2009 guidelines. No statistical differences were found in PPV, which was 68.6% (55.0–79.7) using DETECT compared to 88.9% (74.7–95.6) using the ESC/ERS 2009 guidelines. However, NPV reached 100% (75.7–100) using the DETECT algorithm, while using the ESC/ERS 2009 guidelines it was 88.9% (71.9–96.1).

**Table 4:** Comparisons of the DETECT algorithm and the ESC/ERS 2009 guidelines

	PAH vs no PH patients (*n* = 63)			Patients with mPAP ≥21 vs mPAP <21mmg (*n* = 63)			PH vs no PH patients (*n* = 83)		
	PAH (*n* = 35)	No PH (*n* = 28)	*P* value	mPAP ≥21 mmHg (*n* = 49)	mPAP <21mmg (*n* = 14)	*P* value	PH (*n* = 52)	No PH (*n* = 31)	*P* value
DETECT algorithm									
Score step 1^b^	330.0 (±25.3)	313.7 (±17.5)	<0.01	326.9 (±22.8)	308.6 (±20.8)	<0.01	327.9 (±23.6)	311.8 (±17.6)	<0.01
Indication of Echo^a^	35 (100)	24 (85.7)	0.03	49 (100)	10 (71.4)	<0.01	51 (98.1)	24 (77.4)	<0.01
Score step 2^b^	60.2 (±11.3)	37.3 (±8.4)	<0.001	54.7 (±13.3)	33.5 (±8.8)	<0.001	56.4 (±12.4)	36.5 (±8.4)	<0.001
RHC recommendation^a^	35 (100)	16 (57.1)	<0.001	46 (93.9)	5 (35.7)	<0.001	51 (98.1)	17 (54.8)	<0.001
ESC/ERS 2009 guidelines									
RHC recommendation^a^	32 (91.4)	4 (14.3)	<0.001	34 (69.4)	2 (14.3)	<0.001	42 (80.8)	4 (12.9)	<0.001

*PAH* pulmonary arterial hypertension, *PH* pulmonary hypertension, *mPAP* mean pulmonary artery pressure, *Echo* echocardiography, *RHC* right heart catheterization, *ESC/ERS* European Society of Cardiology and the European Respiratory Society. ^a^Data are shown as number (%) for categorical variables. ^b^Data are shown as mean (± SD)

**Table 5:** Observation of PAH detection programs

		RHC referral rate, %	Missed diagnoses, %	Sensitivity %	Specificity %	PPV %	NPV %
PAH vs no PH patients	DETECT algorithm (95% CI)	51/63, 80.9% -	0/35, 0% -	100% (90.1–100)	42.9% (26.5–60.9)	68.6% (55.0–79.7)	100% (75.7–100)
	ESC/ERS guidelines (95% CI)	36/63, 57.1% -	3/35, 8.5% -	91.4% (77.6–97.0)	85.7% (68.5–94.3)	88.9% (74.7–95.6)	88.9% (71.9–96.1)
Patients with mPAP ≥21 vs mPAP <21mmg	DETECT algorithm (95% CI)	51/63, 80.9% -	3/49, 6.1% -	93.9% (83.5–97.9)	64.3% (38.8–83.7)	90.2% (79.0–95.7)	75.0% (46.8–91.1)
	ESC/ERS guidelines (95% CI)	36/63, 57.1% -	15/49, 30.6% -	69.4% (55.5–80.5)	85.7% (60.1–96.0)	94.4% (81.9–98.5)	44.4% (27.6–62.7)
PH vs no PH patients	DETECT algorithm (95% CI)	68/83, 81.9% -	1/52, 1.9% -	98.1% (89.9–99.7)	45.2% (29.2–62.2)	75.0% (63.6–83.8)	93.3% (70.2–98.8)
	ESC/ERS guidelines (95% CI)	46/83, 55.4% -	10/52, 19.2% -	80.8% (68.1–89.2)	87.1% (71.1–94.9)	91.3% (79.7–96.6)	73.0% (57.0–84.6)

Data shown as number (%). *RHC* right heart catheterization, *PPV* positive predictive value, *NPV* negative positive value, *PAH* pulmonary arterial hypertension, *PH* pulmonary hypertension, *CI* confidence interval, *mPAP* mean pulmonary artery pressure, *ESC/ERS* European Society of Cardiology and the European Respiratory Society

As a strategy to identify mPAP ≥21 mmHg (both BoPAP and PAH), the DETECT algorithm had superior sensitivity of 93.9% (83.5–97.9) and NPV of 75.0% (46.8–91.1) compared to ESC/ERS guidelines for which the sensitivity and NPV was 69.4% (55.5–80.5) and 44.4% (27.6–62.7), respectively. Furthermore, DETECT missed 6.1% of diagnoses of mPAP ≥21, whereas using the ESC/ERS 2009 guidelines the percentage was increased to 30.6%. In fact, analysing only patients with BoPAP, there were 3 out of 14 (21.1%) missed diagnoses using DETECT, whereas there were 12 out of 14 (85.7%) missed using the ESC/ERS 2009 guidelines. However, specificity and PPV were slightly lower using the DETECT algorithm, at 64.3% and 90.2% compared to 85.7% and 94.4%, respectively, using the ESC/ERS guidelines.

To investigate the capacity for the detection of any type of PH, the whole cohort of patients (*n* = 83) with a first RHC were studied, regardless of whether they met the inclusion or exclusion criteria for application of the DETECT algorithm: 1.9% of diagnoses of PH were missed using the DETECT algorithm compared with 19.2% missed using the ESC/ERS 2009 guidelines. Moreover, sensitivity and NPV were greater at 98.1% and 93.3% using the DETECT method, compared with 80.8% and 73.0%, respectively, using the ESC/ERS guidelines. Nevertheless, specificity and PPV were again slightly inferior at 45.2% and 75.0% when applying the DETECT algorithm, whereas the values were 87.1% and 91.3%, respectively, using in the ESC/ERS guidelines.

## Discussion

The present retrospective study compared the application of the DETECT algorithm with the ESC/ERS 2009 guidelines in patients with PAH and patients without PH, who had undergone RHC. The DETECT programme had greater sensitivity, the RHC would have been recommended in all patients suffering from PAH, and also superior NPV compared to the ESC/ERS guidelines. Nevertheless, the ESC/ERS 2009 guidelines had higher specificity and PPV than the DETECT algorithm. Furthermore, the DETECT algorithm had higher percentage sensitivity and NPV both for the detection of patients with mPAP ≥21 and for patients with any type of PH, although specificity and PPV were lower.

For the application of the DETECT algorithm it is necessary to meet the inclusion criteria, so patients must present with a duration of SSc longer than 3 years from the first non-RP symptom and DLCO <60% predicted. This was to increase the specificity of the screening method, due to the fact that <10% of a cohort of 243 patients with SSc-PH have been found to present with DLCO >60% [19]. Furthermore, patients could not fulfil the exclusion criteria such as previous PH diagnosed by RHC, forced vital capacity (FVC) <40% or the involvement of left heart disease [12]. Nevertheless, DETECT does not establish the periodicity of an assessment by this method, it has not been evaluated over a monitoring period in a cohort of patients and its ability to predict mPAP ≥21 mmHg or any type of PH have not been explored. As a consequence of this work, the new ESC/ESR 2015 guidelines were recently published, which strengthen the recommendation for annual screening of PH in this set of patients with SSc and introduce the DETECT algorithm as a valid method to be performed in this group of patients based on the evidence demonstrated [15].

Compared to the study of Coghlan et al., patients in our cohort had a surprisingly higher prevalence of telangiectasias and ACA antibodies. Almost 60% of patients with PAH presented with WHO functional class I or II and mild reduced 6MWD comparable to the detection cohorts of previous studies that have focused on early PAH diagnosis [10, 12]. This fact seems to reflect that patients in our study were referred promptly for RHC as a result of active and early suspicion of PAH during monitoring. In the present paper we describe similar sensitivity, specificity and NPV values, although with a higher RHC referral ratio and PPV using the DETECT algorithm than in the original work. However, regarding the application of ESC/ERS 2009 guidelines we report higher sensitivity, specificity and PPV values and a higher RHC referral ratio than Coghlan et al. This may be explained due to the fact that the main indication for performing RHC in our study was the presence of an abnormal echocardiographic result, suggesting the presence of PAH. Furthermore, the high RHC referral ratio may be justified because the study was conducted in a selected population of patients at high risk of PAH based on clinical, pulmonary function test and echocardiographic parameters.

Few groups have published on the application of the DETECT algorithm. Hao et al. analysed the DETECT algorithm in patients from the multicentre Australian Scleroderma Cohort Study (ASCS) along with the Australian Scleroderma Interest Group (ASIG) algorithm and the ESC/ERS 2009 guidelines [20, 21]. The authors found higher sensitivity and NPV using the DETECT or the ASIG algorithm than they did using the ESC/ERS guidelines, which we can confirm in our study. Surprisingly, the ASIG protocol had superior specificity to the DETECT algorithm and even to the ESC/ERS guidelines, which contrasts with the values of 48% and 69%, respectively, that were previously published [12]. The authors reported that nine patients did not meet the criterion of DLCO <60% or the criterion for disease duration, and this may explain the lower specificity of the DETECT algorithm [21]. Furthermore, the DETECT and ASIG screening methods had the highest sensitivity and specificity to identify both precapillary PH and any group of PH, results that we have corroborated in our work. In the present work the ASIG programme was not analysed due to the fact that this method is not based on evidence and has not been well-validated in European cohorts.

Recently, a Czech scleroderma group designed a modified DETECT algorithm, in which a right ventricular outflow tract is evaluated instead of RA area [22]. Using this modified DETECT programme the RHC referral ratio was 41.4% compared to 24.1% using the ESC/ERS guidelines. Interestingly, although only 11/58 patients (18.9%) had undergone RHC, in patients who were recommended by the modified DETECT algorithm there were also other types of PH diagnosed, such as PH due to left heart disease and PH due to ILD. Therefore, this article suggests that the DETECT algorithm would be useful for recommending RHC for diagnosing other types of PH according to the WHO classification.

Although there are some discrepancies on the prognostic role of BoPAP, a study has been recently published evidencing a higher risk of mortality and hospitalization in US veteran patients with BoPAP (defined as 19–24 mmHg) [23]. Consequently, the study highlights the relevance of diagnosing BoPAP in selected groups of patients such as those with scleroderma. In a post-hoc analysis of the DETECT study, the authors demonstrated that patients with BoPAP had a higher FVC/DLCO rate, and higher TRV and NT-proBNP levels than in patients with normal mPAP [16]. Although the paper does not evaluate the application of DETECT in both groups of patients, it would support the increment of the specificity of this programme identified in our work when studying patients with mPAP ≥21 versus mPAP <21mmg, compared to the PAH versus no PH strategy. In the set of patients with SSc, identifying BoPAP in the first RHC was demonstrated as a high risk factor for developing PH, with a hazard ratio of 3.7 during follow up compared to patients with mPAP <21 mmHg [24]. In this line, in a multicentre prospective SSc cohort, the Pulmonary Hypertension Assessment and Recognition of Outcomes in Scleroderma (PHAROS) study group reported that 55% of patients with BoPAP progressed to PH, whereas in the group of patients with mPAP <21 mmHg this was 32%, over a mean 25.7-month period of monitoring [25]. Having established the relevance of identifying patients with SSc and BoPAP for close supervision, this is the first paper that demonstrates the accuracy of the DETECT algorithm in the identification of patients with mPAP ≥21.

The main limitation of the present study was the retrospective design. RHC was not performed in the whole cohort of patients with SSc, therefore some mild forms of PAH may have been undiagnosed. In the same line, although the periodicity of performing the screening methods is not well established, these were not carried out in the whole cohort, which may not reflect the real referral rate for RHC.

## Conclusions

Pulmonary arterial hypertension in one of the leading causes of death in patients with SSc, and its early diagnosis has demonstrated increased survival rates. The present study supports the DETECT algorithm as an excellent screening method due to its high sensitivity and NPV, which minimizes the number of missed diagnoses of previous recommendations. Furthermore, our data suggest the DETECT algorithm is also accurate either for an early diagnosis of borderline mPAP, patients who are at highest risk of developing PAH, or the diagnosis of any type of PH.

## Abbreviations

6MWD: 6-Minute walking distance
ACA: Anticentromere antibodies
ACR: American College of Rheumatology
ANA: Antinuclear antibodies
ASCS: Australian Scleroderma Cohort Study
ASIG: Australian Scleroderma Interest Group
BoPAP: Borderline median pulmonary arterial pressure
CI: Confidence interval
CO: Cardiac output
dcSSc: Diffuse cutaneous systemic sclerosis
DLCO: Diffusing capacity for carbon monoxide
DU: Digital ulcers
ECG: Electrocardiography
ERA: Endothelin receptor antagonist
ESC/ERS: European Society of Cardiology and the European Respiratory Society
EULAR: European League Against Rheumatism
FVC: Forced vital capacity
HR: Hazard ratio
HRCT: High-resolution computed tomography
IIF: Indirect immunofluorescence
ILD: Interstitial lung disease
IQR: Interquartile range
IV: Intravenous
lcSSc: limited cutaneous systemic sclerosis
LHD: Left heart disease
LVEF: Left ventricular ejection fraction
mPAP: Median pulmonary arterial pressure
NPV: Negative predictive value
NT-proBNP: N-terminal pro-brain natriuretic peptide
PAH: Pulmonary arterial hypertension
PAWP: Pulmonary artery wedge pressure
PDE5 I: Phosphodiesterase type-5 inhibitor
PFT: Pulmonary function test
PH: Pulmonary hypertension
PHAROS: Pulmonary Hypertension Assessment and Recognition of Outcomes in Scleroderma
PPV: Positive predictive value
PVR: Pulmonary vascular resistance
RA: Right atrium
RAP: Right atrial pressure
RHC: Right heart catheterization
RP: Raynaud’s phenomenon
RVSP: Right ventricular systolic pressure
SaO2: Arterial oxygen saturation
SD: Standard deviation
SRC: Scleroderma renal crisis
SSc: Systemic sclerosis
ssSSc: Sine scleroderma systemic sclerosis
SvO2: Mixed venous oxygen saturation
SVR: Systemic vascular resistance
TAPSE: Tricuspid annular plane systolic excursion
TPG: Transpulmonary pressure gradient
TRV: Tricuspid regurgitant velocity
vs: Versus
WHO: World Health Organization
WU: Wood units
